# The Minimal Effect of Linker Length for Fatty Acid Conjugation to a Small Protein on the Serum Half-Life Extension

**DOI:** 10.3390/biomedicines8050096

**Published:** 2020-04-26

**Authors:** Jinhwan Cho, Junyong Park, Giyoong Tae, Mi Sun Jin, Inchan Kwon

**Affiliations:** 1 School of Materials Science and Engineering, Gwangju Institute of Science and Technology (GIST), Gwangju 61005, Korea; kroea2002@gist.ac.kr (J.C.); gytae@gist.ac.kr (G.T.); 2 Department of Biomedical Science and Engineering, GIST, Gwangju 61005, Korea; happydragon@gist.ac.kr; 3 School of Life Science, GIST, Gwangju 61005, Korea; misunjin@gist.ac.kr

**Keywords:** serum half-life extension, fatty acid conjugation, FcRn-mediated recycling, serum albumin

## Abstract

Conjugation of serum albumin or one of its ligands (such as fatty acid) has been an effective strategy to prolong the serum half-lives of drugs via neonatal Fc receptor (FcRn)–mediated recycling of albumin. So far, fatty acid (FA) has been effective in prolonging the serum half-lives for therapeutic peptides and small proteins, but not for large therapeutic proteins. Very recently, it was reported a large protein conjugated to FA competes with the binding of FcRn with serum albumin, leading to limited serum half-life extension, because primary FA binding sites in serum albumin partially overlap with FcRn binding sites. In order to prevent such competition, longer linkers between FA and the large proteins were required. Herein, we hypothesized that small proteins do not cause substantial competition for FcRn binding to albumin, resulting in the extended serum half-life. Using a small protein (28 kDa), we investigated whether the intramolecular distance in FA-protein conjugate affects the FcRn binding with albumin and serum half-life using linkers with varying lengths. Unlike with the FA-conjugated large protein, all FA-conjugated small proteins with different linkers exhibited comparable the FcRn binding to albumin and extended serum half-life.

## 1. Introduction

Therapeutic proteins have been widely used for the treatment of human diseases [1]. However, therapeutic proteins are rapidly eliminated from the blood of patients due to several mechanisms, including renal filtration, proteolysis, intracellular degradation, and immune responses, which result in short serum half-lives [2,3]. Such short serum half-lives of therapeutic proteins result in the need for frequent administration [2,3,4]. Therefore, the extension of the serum half-life of therapeutic proteins is very important in developing new therapeutic proteins [2,4,5]. Conventionally, polyethylene glycol (PEG) chains have been conjugated to therapeutic proteins for serum half-life extension in order to reduce renal filtration and proteolysis [6]. However, recently several concerns have been raised about PEG conjugation, including PEG accumulation due to its poor degradability in the body and reduced efficacy resulting from immune responses to PEG [7].

As an alternative to PEG, human serum albumin (HSA) has been investigated as a half-life extender [8]. In contrast to PEG, HSA is biodegradable and minimally immunogenic [9,10]. Furthermore, HSA has an exceptionally long serum half-life (more than two weeks) partly due to evasion from intracellular degradation via neonatal Fc receptor (FcRn)–mediated recycling [2,3,10,11,12,13]. Direct coupling of therapeutic protein to HSA via either genetic fusion or chemical conjugation led to a significant extension of therapeutic protein serum half-life in vivo [11,12,14,15,16,17]. Recently, indirect coupling of therapeutics to HSA using albumin ligands such as fatty acids has received much attention, because fatty acids are cheaper than HSA and exhibit a much higher conjugation yield than HSA [9]. Furthermore, fatty acids are biocompatible and not immunogenic [18].

**Figure 1:**
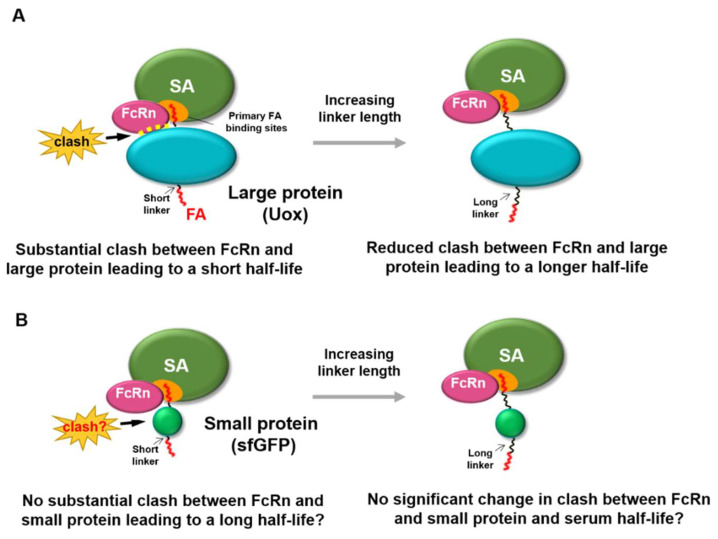
Effect of protein size of fatty acid-conjugated protein on the binding of FcRn with serum albumin. (**A**) For a large protein (Uox, 140 kDa), conjugation of fatty acid with a short linker leads to steric hindrance to binding of FcRn to serum albumin, due to its large size. Increasing the linker length reduces the steric hindrance to binding of FcRn to serum albumin, resulting in longer serum half-life. (**B**) For a small protein (superfolder green fluorescent protein [sfGFP], 28 kDa), conjugation of a fatty acid with a short linker may not exhibit substantial steric hindrance to binding of FcRn to serum albumin, due to its small size. Therefore, increasing linker length may not substantially alter the steric hindrance to the binding of FcRn to serum albumin.

Despite the numerous reports of serum half-life extension via fatty acid conjugation, its molecular mechanism was not yet fully revealed. It is noteworthy that fatty acid conjugation was effective for serum half-life extension of therapeutic peptides and small proteins (less than 28 kDa) [17,19,20,21,22,23,24]. Considering that a majority of therapeutic proteins and other candidates have molecular weights greater than 28 kDa [25], it is important to understand whether there is any protein size–dependent factor in serum half-life extension via fatty acid conjugation. Regarding this, a very recent report showed that fatty acid conjugation extends the serum half-life of urate oxidase (Uox) (140 kDa) [26], a large therapeutic protein used to treat hyperuricemia [27,28,29,30,31]. In this report, the conventional short linker between fatty acid (palmitic acid) and large protein (Uox) was shown not to be able to substantially extend the serum half-life of Uox, due to compromised FcRn binding to serum albumin. In order to avoid competition with FcRn binding and achieve the substantial serum half-life extension, longer linkers between fatty acid and Uox were required (Figure 1A) [26]. There are seven palmitic acid (PA) binding sites on serum albumin (FA1 to FA7) [32]. Two dominant PA binding sites at domain III of serum albumin (FA4 and FA5) [33,34,35,36,37,38] overlap with FcRn binding sites. With a short linker, PA-conjugated Uox (Uox-PA) was expected to be located near domain III of serum albumin. Therefore, the existence of bulky Uox near domain III was attributed to the significant reduction of FcRn binding to serum albumin in vitro, resulting in limited extension in serum half-life in vivo [26]. In contrast, longer linkers were expected to locate Uox-PA away from domain III, leading to the recovery of FcRn binding affinity to serum albumin in vitro and substantial extension of serum half-life in vivo [26].

Considering that fatty acid conjugation led to significant serum half-life extension of therapeutic peptides and small proteins [17,19,20,21,22,23], we hypothesized that small proteins exhibit minimal steric hindrance to binding of FcRn to serum albumin. Therefore, herein we investigated whether fatty acid conjugation to a small protein with linkers with various lengths affects binding of FcRn with serum albumin in vitro and serum half-life in vivo. For a small protein, if linkers with varying lengths do not substantially affect the binding affinity of FcRn to serum albumin in vitro and serum half-life in vivo (Figure 1B), that would demonstrate that protein size is an important factor in serum half-life extension via fatty acid conjugation. These results would facilitate our understanding of the molecular mechanism of fatty acid conjugation–mediated serum half-life extension of proteins.

## 2. Materials and Methods 

### 2.1. Materials

Ampicillin; palmitic acid *N*-hydroxysuccinimide (NHS-PA); 3,3′,5,5′-tetramethylbenzidine (TMB); sinapinic acid; and deoxycholate (DCA) were obtained from Sigma-Aldrich (St. Louis, MO, USA). Mouse serum albumin (MSA) was obtained from Equitech-Bio Inc. (Kerrville, TX, USA). Isopropyl β-D-1-thiogalactopyranoside (IPTG), amine-binding plate, and phosphate-buffered saline (PBS) were obtained from Thermo Fisher Scientific (Waltham, MA, USA). Tween-20 was obtained from Bio-Rad (Hercules, CA, USA). Nickel-nitrilotriacetic acid (Ni-NTA) agarose beads and polypropylene columns were obtained from Qiagen Inc (Valencia, CA, USA). PD-10 column was obtained from GE Health care (Piscataway, NJ, USA). Vivaspin centrifugal concentrators with molecular weight cut-off (MWCO) of 10 kDa were purchased from Sartorius Corporation (Bohemia, NY, USA). Dibenzocyclooctyne (DBCO)-amine, DBCO-PEG4-amine, azidoacetic acid *N*-hydroxysuccinimide (NHS) ester, and azido-PEG4-NHS ester were obtained from Click Chemistry Tools LLC (Scottsdale, AZ, USA). 6×His tag antibody and horseradish peroxidase (HRP)–conjugated anti-rabbit Immunoglobulin G (IgG) were obtained from Cell Signaling Technology (Beverly, MA, USA). ZipTip with C18 resin was purchased from Millipore Corporation (Billerica, MA, USA). Protein Standard II was obtained from Bruker Daltonics (Bremen, Germany). Mouse FcRn was obtained from ACRO Biosystems (Newark, DE, USA).

### 2.2. Preparation of Purified Superfolder Green Fluorescent Protein (sfGFP) from E. coli

In order to obtain purified sfGFP using *E. coli*, cloning, expression, and purification of sfGFP were conducted as previously described [23]. Briefly, the pQE80-sfGFP plasmid was transformed into TOP10 *E. coli* competent cells. The transformed colony was precultured into 2×·YT medium containing 100 μg/mL ampicillin. After 8 h incubation at 37 °C, the precultured cells were inoculated into a 2×·YT medium containing 100 μg/mL ampicillin and incubated at 37 °C for the main culture. When the optical density at 600 nm reached 0.5, 1 mM IPTG was added to the main culture for sfGFP induction. After 5 h incubation at 37 °C, cells were collected by pellet down at 5000 g for 10 min. The cell pellets were stored at −80 °C until further use. In order to start the purification of sfGFP, the cell pellets were resuspended with lysis buffer (pH 7.4, 10 mM imidazole) by complete vortexing. The resuspended cell pellets were broken down by sonication for 1 h. Cell debris was removed by centrifugation at 12,000 rpm for 30 min. The supernatant was mixed with Ni-NTA agarose beads thoroughly and incubated at 15 °C and 220 rpm for 1 h, then was poured into a polypropylene column, followed by washing with washing buffer (pH 7.4, 20 mM imidazole) to remove impurities. The sfGFP was eluted with elution buffer (pH 7.4, 250 mM imidazole), and then was immediately subjected to buffer exchange into PBS buffer (pH 7.4) using a PD-10 column. Finally, the purified sfGFP was concentrated to a proper concentration with a Vivaspin column (MWCO: 10 kDa) according to the supplier’s manual and stored at 4 °C before use. The molar extinction coefficient at 280 nm value of sfGFP was calculated to be 19,035 M^−1^ cm^−1^ by the following equation: ε_280_ = (5500 × number of tryptophan residues) + (1490 × number of tyrosine residues) + (125 × number of disulfide bonds) [39]. The concentration of sfGFP was then determined using the Beer-Lambert law.

### 2.3. Preparation of sfGFP-PA Conjugates with Various Linkers

**Figure 2:**
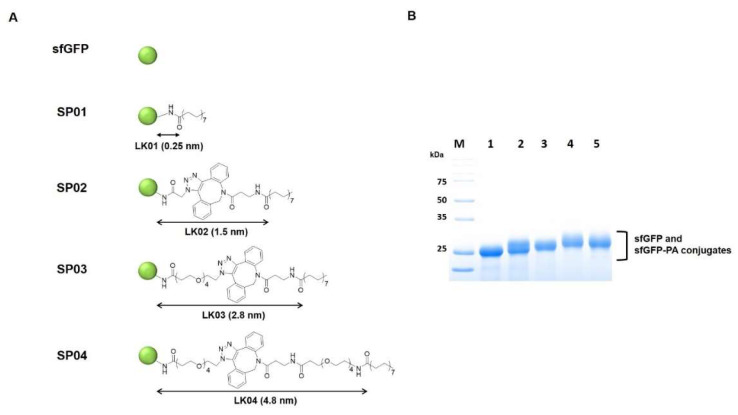
The structures and characterization of sfGFP-PA conjugates with various linker lengths. (**A**) sfGFP is represented as a green circle. The length of each linker was measured between the ε-carbon in a lysine residue of sfGFP and carbonyl carbon of PA when the linker was maximally stretched using Chem3D software and marked with black arrows. (**B**) Coomassie blue–stained protein gel image of purified sfGFP and sfGFP-PA conjugates was taken by Bio-Rad ChemiDoc XRS+. Lane M, molecular weight markers; Lane 1, sfGFP; Lane 2, SP01; Lane 3, SP02; Lane 4, SP03; Lane 5, SP04.

The sfGFP-PA conjugates with various linker lengths were prepared as previously reported, except sfGFP was used instead of Uox [26]. The chemical structures of intermediates and linkers (LK01, 02, 03, and 04) are shown in Figure 2A and Figure S1 in Supplementary Materials. Briefly, each DBCO-amine or DBCO-PEG4-amine (180 μM) was reacted with NHS-PA (900 μM) at 37 °C for 20 h to make DBCO-PA or DBCO-PEG4-PA, respectively. The unreacted NHS-PA was quenched with excess Tris base (100 mM, pH 7.4). The sfGFP-PA conjugates with various linker lengths (SP01, SP02, SP03, and SP04) were generated using three different PA-containing reagents (NHS-PA, DBCO-PA, and DBCO-PEG4-PA). First, sfGFP (50 μM) and NHS-PA (500 μM) were reacted in PBS containing 0.40% (w/v) DCA at room temperature for 3 h, yielding SP01. Second, sfGFP (50 μM) and azidoacetic acid NHS ester (1500 μM) were reacted in PBS on ice for 2 h and quenched with excess Tris base (150 mM, pH 7.4) to make sfGFP-azides intermediate. After desalting and concentration by Vivaspin (MWCO: 10 kDa), the concentration of sfGFP-azides intermediate was measured using the Beer-Lambert law. sfGFP-azides intermediate (50 μM) was reacted with DBCO-PA (100 μM) in PBS with 0.80% (w/v) DCA at room temperature for 3 h, yielding SP02. Third, sfGFP (50 μM) and azido-PEG4-NHS ester (1500 μM) were reacted in PBS on ice for 2 h and quenched with excess Tris base (150 mM, pH 7.4) to make sfGFP-PEG4-azides intermediate. After desalting and concentration by Vivaspin (MWCO: 10 kDa), the concentration of sfGFP-PEG4-azides was measured using the Beer-Lambert law. sfGFP-PEG4-azides (50 μM) was reacted with DBCO-PA (100 μM) in PBS with 0.80% (w/v) DCA at room temperature for 3 h, yielding SP03. Fourth, sfGFP-PEG4-azides (50 μM) was reacted with DBCO-PEG4-PA (100 μM) in PBS with 0.80% (w/v) DCA at room temperature for 3 h, yielding SP04. Finally, all sfGFP-PA conjugates were desalted to PBS buffer (pH 7.4) using a PD-10 column and stored at 4 °C until required for use.

### 2.4. Matrix-Assisted Laser Desorption Ionization/Time-of-Flight (MALDI-TOF) Analysis of the sfGFP and sfGFP-PA Conjugates

In order to analyze the intact mass of the sfGFP or sfGFP-PA conjugate, the sample was desalted on a ZipTip C18 according to the manufacturer’s (Millipore Corporation) protocol. The first layer applied to a polished steel plate using sinapinic acid in absolute ethanol. The desalted sfGFP or sfGFP-PA conjugate was mixed with 1:1 of sinapinic acid in TA30 solution (0.1% Trifluoroacetic acid: acetonitrile = 7:3) and then applied to the first layer prior to mass analysis via Microflex MALDI-TOF (Bruker Daltonics; Bremen, Germany). The MALDI-TOF was calibrated using a Protein Standard II (20–90 kDa) before measurement according to the manufacturer’s instructions. The average masses of sfGFP, SP01, SP02, SP03, and SP04 were obtained by multiplying each area and its corresponding mass of all peaks and then dividing its average value by the average area of all peaks. The average numbers of conjugated PAs at SP01, SP02, SP03, and SP04 were obtained by using the molecular weight of the PA containing linker from the mass shift from sfGFP, sfGFP-azides, and sfGFP-PEG4-azides.

### 2.5. In Vitro Serum Albumin Binding Assay

MSA (10 μg/mL) in 100 μL of PBS (pH 7.4) was applied to amine-binding plates at 4 °C overnight. 5% skim milk in PBS containing 0.05% (v/v) of Tween-20 (PBS-T) (pH 7.4) was used for blocking nonspecific binding at room temperature for 2 h. sfGFP and sfGFP-PA conjugates (14 μM) in 50 μL of PBS were incubated on the plate at room temperature for 2 h. The amount of sfGFP bound was determined by enzyme-linked immunosorbent assay (ELISA) using the anti-6×His antibody. After that, the anti-6×His antibody (1:1000 diluted) was incubated at room temperature for 2 h and washed. Immediately after, HRP-conjugated anti-rabbit IgG (1:1000 diluted) was added and incubated for 1 h. After washing, the TMB substrate was added, incubated for the appropriate time, and stopped with 1 M sulfuric acid. Absorbance at 450 nm was monitored using a Synergy H1 multimode microplate reader. (BioTek; Winooski, VT, USA).

### 2.6. sfGFP Fluorescence Assay

The fluorescence of sfGFP or sfGFP-PA conjugate was measured using a Synergy H1 multimode microplate reader. Each sfGFP or sfGFP-PA conjugate (1.7 μM) in 100 μL of PBS was added to a 96-well plate, and then the fluorescence was measured at an excitation wavelength of 480 nm and an emission wavelength of 510 nm. The relative fluorescence of sfGFP-PA conjugate was normalized by that of sfGFP.

### 2.7. Serum Half-Life Determination in Mice

The amounts of sfGFP and sfGFP-PA conjugate in vivo were investigated by injecting each protein (10 μM) in 200 μL of PBS into the tail veins of 9-week-old female BALB/c mice (n = 5). Experiments on mice were performed according to the guidelines of the Animal Care and Use Committee of the Gwangju Institute of Science and Technology (GIST) (GIST-2019-071). Blood samples (below 50 μL) were collected at 10, 20, 30, 40, and 50 min for sfGFP; 10 min and 1, 2, and 4 h for sfGFP-PA conjugates. Collected blood samples were allowed to clot at room temperature for 30 min, then centrifuged at 1500 rpm for 10 min at 4 °C to obtain the serum from the blood. The separated serum was stored at −20 °C until required for use. The concentrations of sfGFP and sfGFP-PA conjugates in the serum samples were measured by using a GFP ELISA kit according to the manufacturer’s (Cell Biolabs Inc; San Diego, CA, USA) protocol.

### 2.8. FcRn/Serum Albumin/sfGFP-PA Tertiary Complex Formation Assay

Mouse FcRn (10 µg/mL) in 100 µL of PBS (pH 6.0) applied to an amine-binding plate at 4 °C overnight. In order to block nonspecific binding, 5% skim milk in PBS-T (pH 6.0) was added and incubated at room temperature for 2 h. MSA (1 mg/mL) in 100 μL of PBS (pH 6.0) was added at room temperature for 2 h. After washing, 14 µM of sfGFP or sfGFP-PA conjugates in 50 μL of PBS (pH 6.0) was added and incubated at room temperature for 2 h. After that, 100 μL of anti-6×His antibody (1:1000 diluted) was incubated at room temperature for 2 h. After washing, HRP-conjugated anti-rabbit IgG (1:1000 diluted) was added and incubated at room temperature for 1 h. Then, the TMB substrate was added, incubated for the appropriate time, and stopped with 1 M sulfuric acid. Absorbance at 450 nm was monitored with a Synergy H1 multimode microplate reader.

## 3. Results and Discussion

### 3.1. PA-Conjugated sfGFP Conjugates with Various Linker Lengths

We chose sfGFP as a small model protein to investigate the competition to binding of FcRn to serum albumin and the serum half-life extension upon fatty acid conjugation. Although green fluorescent protein is not a therapeutic protein, it has been widely used in biomedical applications [40,41,42,43,44]. In particular, its unique spectral properties including fluorescence and biocompatibility, make sfGFP a great surrogate for a therapeutic protein in drug delivery studies [41,42]. In order to investigate the protein-size dependency of serum half-life extension via fatty acid conjugation, a model protein should be much smaller than Uox (140 kDa). Considering that the molecular weight of sfGFP with an anti-hexahistidine (6×His) tag used in this study is about 28 kDa, sfGFP was suitable as a small model protein. Purified sfGFP was prepared as described previously [8,23,45]. Briefly, the gene of sfGFP was overexpressed in TOP 10 *E. coli* cells. Then, the recombinant sfGFP was purified via metal affinity chromatography using its 6×His tag. The band of purified sfGFP was observed in the Coomassie blue–stained protein gel (Figure 2B, lane 1), indicating that the purity of sfGFP was greater than 95%.

Next, four linkers with various lengths (LK01, 02, 03, and 04) were conjugated to sfGFP to generate sfGFP-PA conjugates (SP01, 02, 03, and 04) (Figure 2A). The same four linkers were previously used to prepare Uox-PA conjugates [26]. In general, properties of the fatty acid linker, such as solubility and length, may affect serum albumin binding affinity. However, for Uox-PA conjugates, the linkers did not exhibit substantial differences in serum albumin binding affinity [26]. Therefore, in order to minimize the effects of fatty acid linkers on serum albumin binding, we chose the same set of linkers for sfGFP-PA conjugates. The lengths of linkers were estimated as 0.25, 1.5, 2.8, and 4.8 nm, respectively [26]. Since these linker lengths were estimated at a maximal stretch, the actual linker lengths could be shorter than those theoretical values. The four sfGFP-PA conjugates (SP01, 02, 03, and 04) were prepared similarly to those of Uox-PA [26]. Briefly, for SP01, NHS-PA (Figure S1a in Supplementary Materials) was directly conjugated to lysine residues of sfGFP via NHS-amine reaction (Figure S1b in Supplementary Materials). In the case of SP02, azidoacetic acid NHS ester (Figure S1c in Supplementary Materials) was reacted with lysine residues of sfGFP to generate sfGFP-azides. DBCO-amine (Figure S1d in Supplementary Materials) was reacted with NHS-PA to generate an intermediate (DBCO-PA) (Figure S1e in Supplementary Materials). Then, DBCO-PA was reacted with sfGFP-azides via strain-promoted alkyne-azide cycloaddition (SPAAC) reactions (Figure S1f in Supplementary Materials) to obtain SP02. In the case of SP03, azido-PEG4-NHS (Figure S1g in Supplementary Materials) was reacted with lysine residues of sfGFP to generate sfGFP-PEG4-azides. Then, DBCO-PA was reacted with sfGFP-PEG4-azides to generate SP03. Finally, for SP04, NHS-PA was reacted with DBCO-PEG4-amine (Figure S1h in Supplementary Materials) to generate DBCO-PEG4-PA (Figure S1i in Supplementary Materials). Then, DBCO-PEG4-PA was reacted with sfGFP-PEG4-azides to generate SP04.

PA conjugation to the four sfGFP-PA conjugates was verified by protein band shifts in the protein gel and mass shifts in MALDI-TOF mass spectra (Figure 2B and Figure S2 in Supplementary Materials). In the protein gel, the bands of sfGFP-PA conjugates (SP01, 02, 03, and 04) were shifted up from the band of unmodified sfGFP, indicating the mass of sfGFP increased upon PA conjugation. Furthermore, we performed a MALDI-TOF spectrometric analysis of sfGFP and sfGFP-PA conjugates. The mass of unmodified sfGFP determined by MALDI-TOF analysis was 27,705 Da, which was consistent with its theoretical value (27,604 Da), with a 0.37% error. For sfGFP-PA conjugates, peaks were right-shifted, indicating that the mass of sfGFP increased upon PA conjugation. For all four sfGFP-PA conjugates, the mass differences between sfGFP-PA conjugate and unmodified sfGFP indicated that the average number of PA conjugated to single sfGFP was about one. Therefore, we speculated that the property differences among sfGFP-PA conjugates resulting from the different number of conjugated PAs were minimal.

### 3.2. Serum Albumin Binding Affinity of sfGFP-PA Conjugates

**Figure 3:**
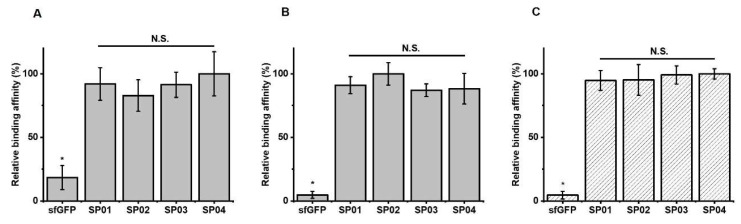
Relative albumin binding affinities of sfGFP-PA conjugate and tertiary complex formation of FcRn/MSA/sfGFP-PA conjugates. The amount of sfGFP or sfGFP-PA conjugate bound on MSA without mouse FcRn at pH 7.4 (**A**) or pH 6.0 (**B**). The amount of sfGFP or sfGFP-PA conjugate complexed with MSA and mouse FcRn at pH 6.0 (**C**). The amount of sfGFP or sfGFP-PA conjugate bound on the plate was normalized based on the highest signal to calculate relative binding affinity. The graph represents the mean ± standard deviations (SD) (n = 3). * *p* < 0.01; N.S.: not significant (two-tailed student *t*-test).

Using the sfGFP-PA conjugates, we first performed serum albumin binding assays at pH 7.4. 96-well plate was coated with MSA. Then, either the purified sfGFP-PA conjugate or unmodified sfGFP was incubated in a well. The amount of either sfGFP-PA conjugate or unmodified sfGFP bound MSA on the plate was analyzed by ELISA using anti-6×His antibody. The binding affinities sfGFP-PA conjugates (SP01, 02, 03, and 04) were comparable but significantly greater than that of unmodified sfGFP (Figure 3A). These results indicate that PA conjugation to sfGFP retained the albumin binding capacity. Furthermore, although linker properties may affect serum albumin binding affinity, the set of linkers used in this study did not substantially alter serum albumin binding affinity for all sfGFP-PA conjugates; this was consistent with the results for Uox-PA conjugates [26].

### 3.3. Fluorescence of sfGFP-PA Conjugates

Next, we investigated whether PA conjugation to sfGFP affected the fluorescence intensity of sfGFP. All sfGFP-PA conjugates (SP01, SP02, SP03, and SP04) exhibited about 20% reduction in fluorescence intensity compared to that of sfGFP (Figure S3 in Supplementary Materials). However, the fluorescence intensities of all sfGFP-PA conjugates were comparable. Such a reduction in fluorescence intensity is likely attributable to the use of DCA to increase the solubility of PAs during conjugation reaction [9,26]. Since DCA is a well-known detergent, it may perturb the folded structure of sfGFP, resulting in reduced fluorescence. Such a moderate reduction in fluorescence intensity of sfGFP-PA conjugate was not problematic in determining the serum half-life of sfGFP-PA because relative residual amounts of sfGFP-PA in serum were analyzed.

### 3.4. Serum Half-Lives of sfGFP-PA Conjugates

**Figure 4:**
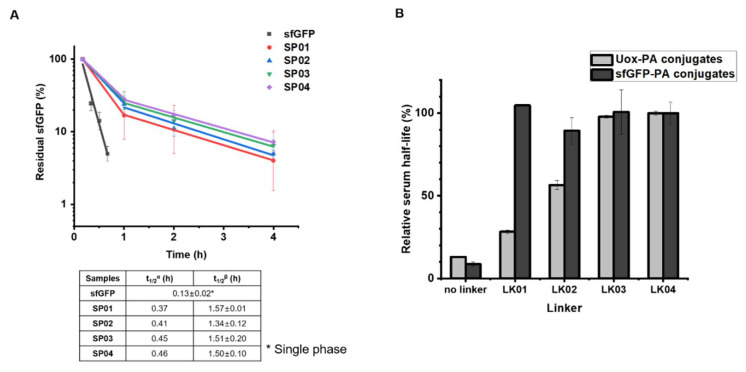
Pharmacokinetic studies of sfGFP and sfGFP-PA conjugates in mice. (**A**) Either purified sfGFP or sfGFP-PA conjugate was intravenously injected into the tail vein of a female Balb/c mouse (n = 4). The concentrations of sfGFP and sfGFP-PA conjugates were measured from blood samples taken at different time points: 10, 20, 30, 40, and 50 min for sfGFP; 10 min and, 1, 2, and 4 h for sfGFP-PA conjugates. The residual sfGFP amounts in serum were plotted on a logarithmic scale. Each data point represents the mean ± SD (n = 4). t_1/2_^α^ and t_1/2_^β^ indicate the serum half-lives in the α- and β-phase, respectively. The serum half-lives of sfGFP and sfGFP-PA conjugates are summarized in the below table. (**B**) Comparison of relative serum half-lives of either Uox-PA (light gray) or sfGFP-PA conjugates (dark gray) versus corresponding linkers. The serum half-lives of unmodified protein and PA conjugates were normalized using those of PA conjugates with the LK04 linker. The serum half-life data of Uox and Uox-PA conjugates were obtained from the literature [26].

Then, the half-lives of sfGFP and sfGFP-PA conjugates were determined from the studies on mice. As reported previously [45], the percentages of residual sfGFP amounts were fitted to a two-phase model (Figure 4A). It was reported that FcRn-mediated recycling affects beta-phase in pharmacokinetics [46]. Therefore, beta-phase half-lives were plotted for sfGFP or sfGFP-PA conjugates in the order of increasing linker length. The half-life of sfGFP (single-phase) was 0.13 h. The beta-phase half-lives of sfGFP-PA conjugates were between 1.34 and 1.57 h, more than 10 times longer than that of sfGFP. This trend was notably different from that of Uox-PA conjugates [26]. In the case of Uox-PA conjugates up to a certain linker length, the half-life of Uox-PA conjugate increased as linker length increased (Figure 4B, light gray). However, the half-lives of sfGFP-PA conjugates were comparable, demonstrating that all linkers allow effective half-life extension of sfGFP in vivo (Figure 4B, dark gray). These results support our hypothesis that, for a small protein such as sfGFP, the distance between PA and small protein does not substantially alter serum half-life extension in vivo.

### 3.5. FcRn Binding Assays of sfGFP-PA Conjugates

In order to investigate whether sfGFP-PA conjugates compete with the binding of FcRn to serum albumin depending on linker length, we performed FcRn binding assays. The assays for sfGFP-PA conjugates were based on the formation of FcRn/serum albumin/sfGFP-PA tertiary structure, as reported previously for Uox-PA conjugates [26]. The dissociation constant between mouse FcRn and MSA was reported to be 546 nM [47]. Therefore, the tertiary structure formation should be dependent of binding of sfGFP-PA with MSA. The pH value of 6.0 was often used to confirm the interaction of albumin with FcRn [45,48]. Hu et al. reported that endo-lysosomal pH varies from 4.5 to 6.5 [49]. Therefore, we chose pH 6.0 for FcRn binding assays in our study. In order to evaluate albumin binding affinities of sfGFP-PA conjugates, the amount of sfGFP-PA bound on MSA was first determined. The albumin binding affinities of all four sfGFP-PA conjugates were comparable at pH 6.0 but significantly greater than that of unmodified sfGFP (Figure 3B). Then, we performed the FcRn binding assays. As expected for a small protein, all sfGFP-PA conjugates (SP01, 02, 03, and 04) showed the comparable formation of FcRn/MSA/sfGFP-PA tertiary complex (Figure 3C), indicating that none of the sfGFP-PA conjugates substantially interfere with FcRn binding to serum albumin in vitro. Combined with the FcRn/serum albumin/sfGFP-PA tertiary complex formation results, the sfGFP-PA conjugate, even with a short linker, does not hinder the FcRn binding to serum albumin; the result is effective half-life extension in vivo. Therefore, the results of sfGFP-PA conjugates support our previous results: Uox requires a long linker in the PA conjugate in order to achieve effective half-life extension in vivo for mitigating the steric hindrance between Uox and FcRn due to its large size. In view of the pharmacokinetics and FcRn binding assay results of large protein Uox-PA conjugates [26] and small protein sfGFP-PA conjugates, it can be concluded that the protein size is the dominating factor to consider for fatty acid conjugation.

## 4. Conclusions

In conclusion, our results showed that, for a small protein (sfGFP, 28 kDa), PA conjugation does not compromise the binding of FcRn to serum albumin in vitro or the serum half-life extension in vivo regardless of linker length. Combined with the previous results for a large protein (Uox, 140 kDa) [24], our results supported that the protein size in the fatty acid–protein conjugate is an important factor for effective binding of FcRn with serum albumin leading to prolonged serum half-life in vivo. We believe our findings can contribute to the successful design of other fatty acid–conjugated therapeutic proteins for serum half-life extension.

## Supplementary Materials

The following are available online at https://www.mdpi.com/2227-9059/8/5/96/s1. Figure S1: The chemical structures of linkers and scheme of reactions for linker preparation. Figure S2: MALDI-TOF mass spectra of sfGFP and sfGFP-PA conjugates. Figure S3: Relative fluorescence of sfGFP and sfGFP-PA conjugates.
