# Supplementary material for: The Minimal Effect of Linker Length for Fatty Acid Conjugation to a Small Protein on the Serum Half-Life Extension
Source: Biomedicines. 2020 Apr 26;8(5):96. doi: 10.3390/biomedicines8050096 (PMC7277390; doi:10.3390/biomedicines8050096)
Supplement: Supplementary file 1 [file biomedicines-08-00096-s001.pdf]

biomedicines

Supplementary Materials: The Minimal Effect of Linker Length for Fatty Acid Conjugation to a Small Protein on the Serum Half-Life Extension

Jinhwan Cho, Junyong Park, Giyoong Tae, Mi Sun Jin, and Inchan Kwon

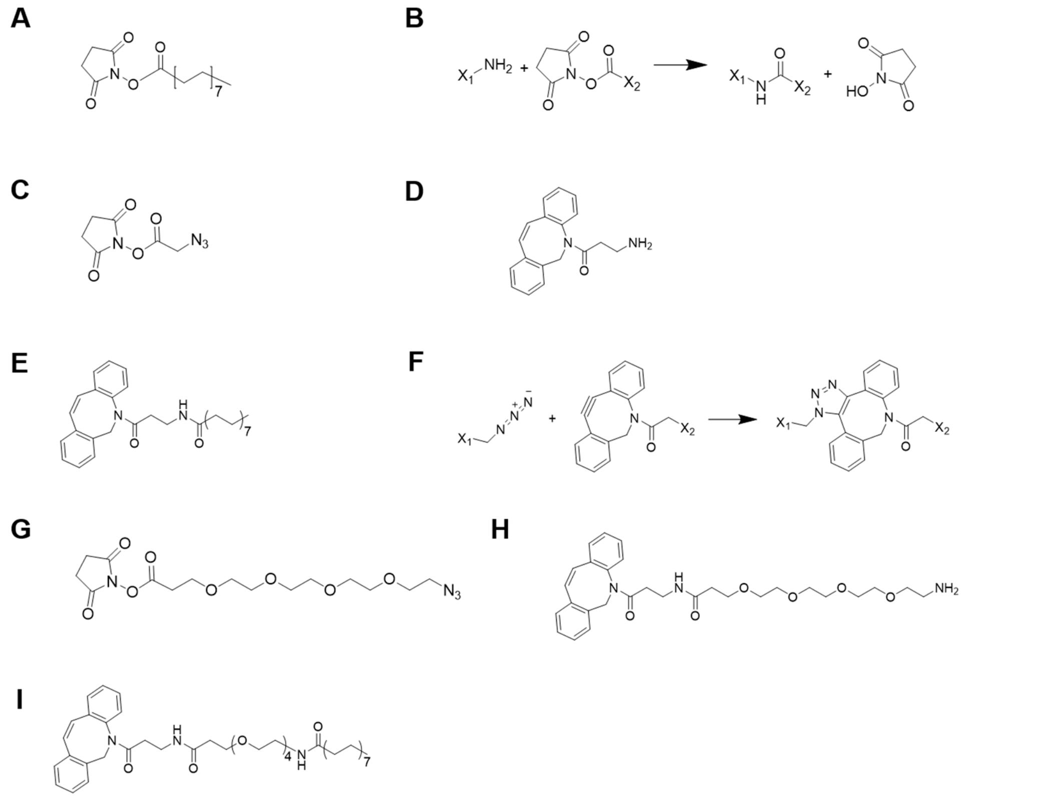

Figure S1. The chemical structures of linkers and schemes of reactions for linker preparation. (**A**) palmitic acid N-hydroxysuccinimide (NHS) ester; (**B**) NHS-amine reaction; (**C**) azidoacetic acid NHS; (**D**) Dibenzocyclooctyne (DBCO)-amine; (**E**) DBCO-palmitic acid; (**F**) strain promoted azide-alkyne cycloaddition (SPAAC); (**G**) azido-PEG4-NHS; (**H**) DBCO-PEG4-amine; (**I**) DBCO-PEG4-palmitic acid. All structures were drawn with ChemDraw software.

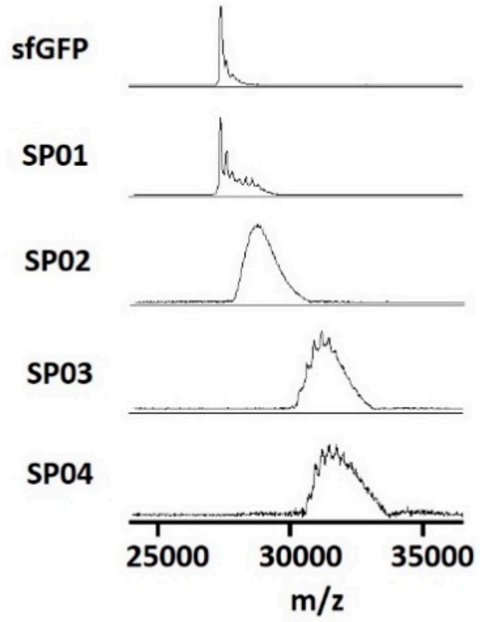

sfGFP variant	Average mass (m/z)	Average mass shift from that of sfGFP (m/z)	Average number of conjugated palmitic acid
sfGFP	27705	-	-
SP01	27950	245	1.0
SP02	28924	1219	1.0
SP03	31426	3721	1.0
SP04	31730	4025	1.1

Figure S2. MALDI-TOF mass spectra of superfolder green fluorescent protein (sfGFP) and PA-conjugated sfGFP (sfGFP-PA) conjugates. sfGFP was conjugated to PA with linkers of various lengths. Average masses, mass shifts, and numbers of conjugated palmitic acids of sfGFP, SP01, SP02, SP03, and SP04 are summarized in the table below MS spectra.

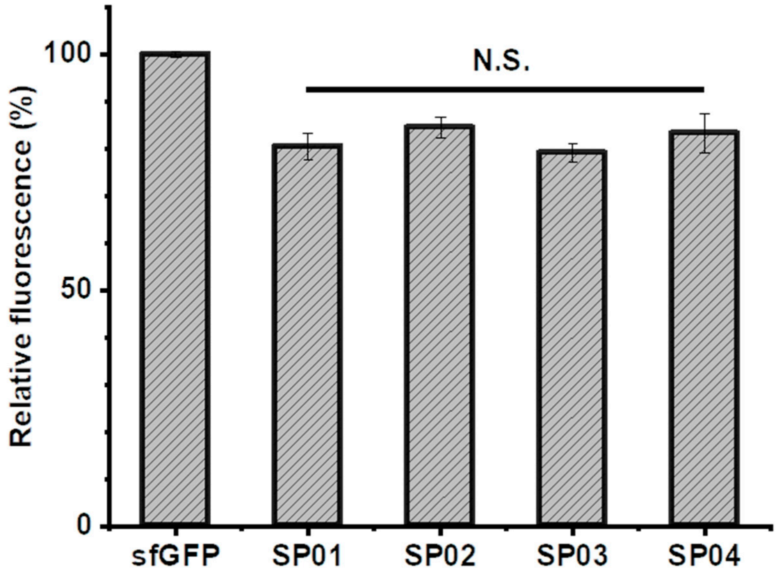

Figure S3. Relative fluorescence of sfGFP and sfGFP-PA conjugates (excitation: 480 nm; emission: 510 nm). The fluorescence of the sfGFP-PA conjugate was normalized by using the fluorescence intensity of sfGFP. The graph represents the mean ± standard deviations (SD) (n = 3). N.S.: not significant (two-tailed student t-test).
